# A new *S.* *suis* serotype 3 infection model in pigs: lack of effect of buprenorphine treatment to reduce distress

**DOI:** 10.1186/s12917-022-03532-w

**Published:** 2022-12-12

**Authors:** Carolin Liedel, Leonie Mayer, Almuth Einspanier, Iris Völker, Reiner Ulrich, Karoline Rieckmann, Christoph G. Baums

**Affiliations:** 1 Institute of Bacteriology and Mycology, Centre for Infectious Diseases, Faculty of Veterinary Medicine, Leipzig University, An den Tierkliniken 29, 04103 Leipzig, Germany; 2 Institute of Physiological Chemistry, Faculty of Veterinary Medicine, Leipzig University, An den Tierkliniken 1, 04103 Leipzig, Germany; 3 Institute of Veterinary Pathology, Faculty of Veterinary Medicine, Leipzig University, An den Tierkliniken 33, 04103 Leipzig, Germany

**Keywords:** *Streptococcus suis*, Experimental infection, Pig, Serotype 3, Refinement, Analgesia, Buprenorphine, Cortisol, Scoring

## Abstract

**Background:**

*Streptoccocus suis* (*S.* *suis*) is a major porcine pathogen causing meningitis, septicemia, arthritis and endocarditis. These diseases severely impair welfare of pigs. Experimental studies in pigs are important to better understand the pathogenesis and to identify protective antigens, as so far there is no vaccine available protecting against various serotypes (*cps*). Due to the severity of disease, application of appropriate refinement strategies in experimental *S.* *suis* infections is essential to reduce distress imposed on the piglets without jeopardizing the scientific output. The objectives of this study were to evaluate buprenorphine treatment as a refinement measure and serum cortisol levels as a distress read out parameter in a new *S.* *suis cps*3 infection model in pigs.

**Results:**

Intravenous application of 2 × 10^8^ CFU of *S.* *suis cps*3 (*sly*^+^, *mrp*^+^) to 6-week-old piglets led to severe morbidity in approximately 50% of the animals. Main pathological findings included suppurative meningoencephalitis and arthritis as well as fibrinosuppurative endocarditis. Buprenorphine treatment (0.05 mg/kg every 8 h) did not prevent signs of severe pain, high clinical scores, moderate to severe pathologies or high levels of serum cortisol in single severely affected piglets. Significant differences in the course of leukocytosis, induction of specific antibodies and bactericidal immunity were not recorded between groups with or w/o buprenorphine treatment. Of note, clinically unobtrusive piglets showed serum cortisol levels at 2 and 5 days post infectionem (dpi) comparable to the levels prior to infection with *cps*3. Cortisol levels in serum were significantly increased in piglets euthanized due to severe disease in comparison to clinically unobtrusive pigs.

**Conclusions:**

Different clinical courses and pathologies are induced after intravenous challenge of piglets with 2 × 10^8^ CFU of this *S.* *suis cps*3 strain. The chosen protocol of buprenorphine application does not prevent severe distress in this infection model. Important parameters of the humoral immune response, such as the level of IgM binding to *S. suis cps*3, do not appear to be affected by buprenorphine treatment. Serum cortisol is a meaningful parameter to measure distress in piglets experimentally infected with *S.* *suis* and to evaluate refinement strategies. In this intravenous model, which includes close clinical monitoring and different humane endpoints, clinics and cortisol levels suggest convalescence in surviving piglets within 5 days following experimental infection.

**Supplementary Information:**

The online version contains supplementary material available at 10.1186/s12917-022-03532-w.

## Background

*Streptococcus suis* (*S.* *suis*) is an invasive pathogen which has received increasing scientific attention because of its zoonotic potential and the emergence as the most important bacterial pathogen in weaning and growing piglets [1]. This pathogen is very diverse as shown by serotyping and analysis of a pairwise distance matrix based on 16S rRNA or chaperonin 60 sequences [2–5]. Substantial differences in virulence between strains of different serotypes (*cps*) but also within certain *cps* such as *cps*2 and 9 have been demonstrated through experimental infections [6, 7]. The capsule of *cps*3 is distinct as it includes the rare diamino sugar di-N-acetyl-d-bacillosamine [8]. Though several in vitro tests [9–13] have been established to read out various immune reactions, experimental infection of pigs is still important in *S. suis* research, e.g. to determine the protective efficacy of a vaccine candidate [14–16]. Although worldwide approximately 15% of reported *S.* *suis* diseases in pigs have been caused by *cps*3 [17], an infection model of this important *cps* in pigs has not been published.

*S. suis* infection might lead to severe clinical signs in association with septicemia, meningitis, arthritis and endocarditis [18, 19]. These diseases cause a substantial amount of distress and pain in pigs. However, analgesic treatment has not been described as a refinement measure in experimental infections of pigs with *S. suis* even though in laboratory animals opioid analgesics are frequently used for pain management [19–21]. Furthermore, they are the first analgesics considered for refinement in immunology models [22] and applied for postoperative analgesia in pigs [23] due to their strong analgesic effect. A discussed disadvantage of opioids is their immunomodulatory effect [24, 25]. However, buprenorphine has turned out to have little side effects on immunological parameters [19, 22]. As a partial receptor agonist, buprenorphine does not increase the release of immunomodulatory glucocorticoids [24] compared to other opioid analgesics. Since buprenorphine is able to reduce pain and lameness in pigs [26, 27] and due to the putative lack of a substantial immunomodulatory effect, we evaluated buprenorphine application post experimental infection as a refinement strategy. The experimental study was designed to read out putative effects of buprenorphine application on the humoral immune response, the distress of the piglets as determined by a clinical as well as a pain score and on cortisol levels in saliva and serum as a distress marker in the course of establishing a new *S.* *suis cps*3 model in pigs.

## Results

### Clinics of *S.* *suis cps*3 infected piglets with or w/o analgesic buprenorphine treatment

**Fig. 1:**
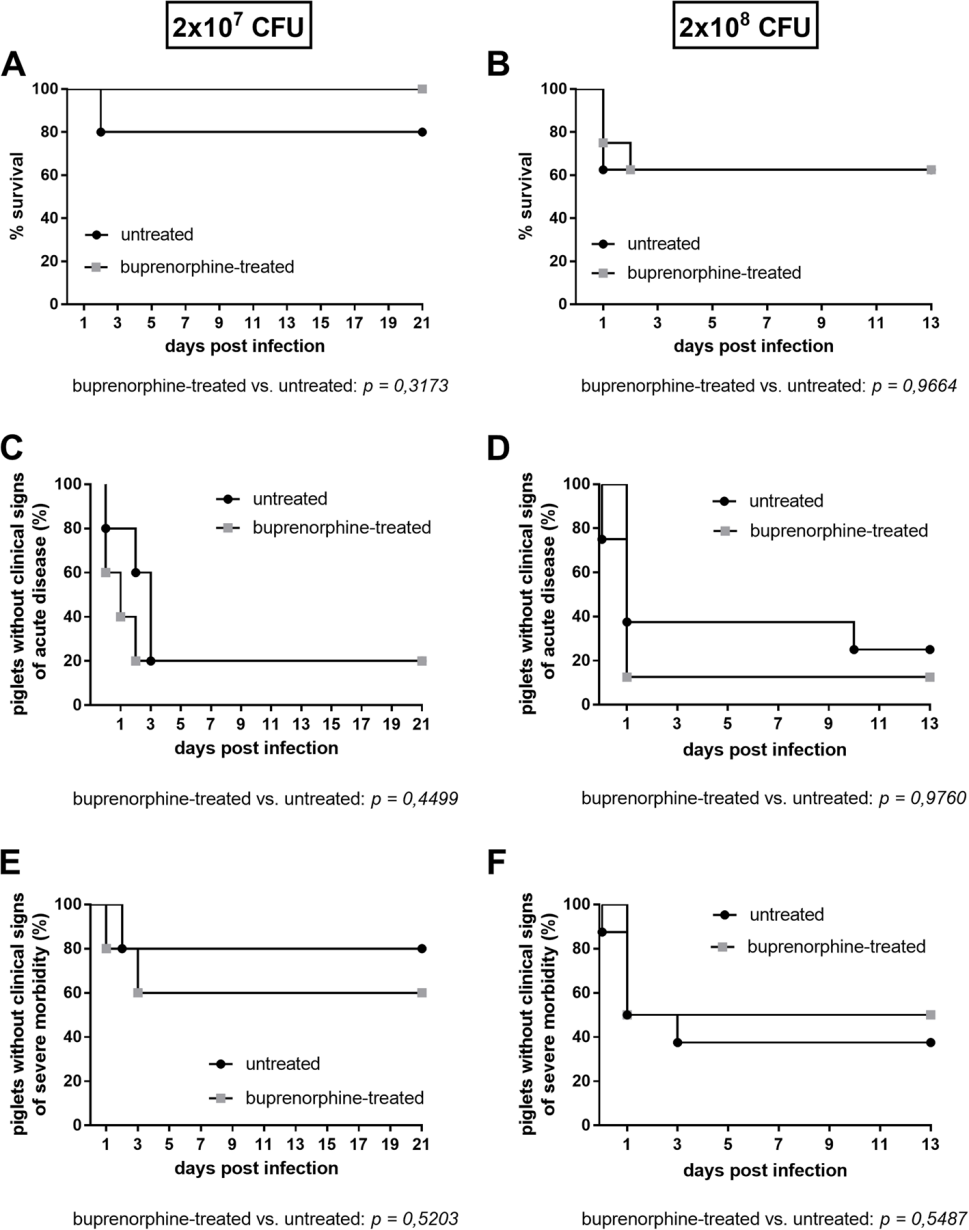
Mortality (**A** + **B**), morbidity (**C** + **D**) and severe morbidity (**E** + **F**) after intravenous *cps*3 infection of 6-week-old piglets. Piglets were infected with 2 × 10^7^ CFU (*n* = 5/group) (**A**; **C**; **E**) or 2 × 10^8^ CFU (*n* = 8/group) (**B**; **D**; **F**) of *S.* *suis cps*3 strain 16667/3. One group was treated with buprenorphine until the 5th day after infection. The health status was monitored every 8 h. Morbidity was defined as inner body temperature ≥ 40.2 °C or acute clinical signs of *S.* *suis* disease. Severe morbidity was defined as inner body temperature of ≥ 40.2˚C in three consecutive controls or an inner body temperature ≥ 40.5˚C and additionally reduced appetite. Piglets were euthanized if pre-defined endpoints such as central nervous disorders or signs of acute polyarthritis or a clinical score ≥ 25 were recorded. Statistical analysis of the Kaplan–Meier diagram was performed using the log-rank test (*p*-values are shown below the diagrams)

Buprenorphine treatment was examined as a refinement strategy in an experimental *S.* *suis* study conducted to establish a new *cps*3 challenge model. We infected 6-week-old piglets intravenously with a *sly*^+^, *mrp*^+^ *cps*3 strain of sequence type (ST) 1187, designated 16667/3. After infection one group received an intramuscular (i.m.) injection of 0.05 mg buprenorphine/kg every 8 h for 5 days. Piglets were monitored every 8 h for clinical signs. Additionally, pain and clinical scores were raised (see Supplementary Tables 1 and 2, Additional file 1). The pain score emphasizes signs of severe distress and pain such as recumbency in association with acute polyarthritis. This score was introduced in this study to disclose, whether piglets infected experimentally with *S.* *suis* may not reach states of severe distress if treated early with buprenorphine. Piglets which showed signs of severe *S.* *suis* disease like polyarthritis, recumbency and central nervous disorder or reached a clinical score ≥ 25 were euthanized due to reaching humane endpoints. In the first experiment with an infection dose of 2 × 10^7^ CFU, mortality was very low. Only one untreated (UT) piglet was humanly euthanized 2 d following intravenous (i.v.) infection due to signs of central nervous disorder like convulsions, tremor and opisthotonus (Fig. 1A). One buprenorphine-treated piglet (BT) showed signs of lameness, tremor and fever after experimental infection. However, as the piglet was still very active and had no anorexia, it did not reach the humane endpoints. In the first experiment 4 (*n* = 3 BT; *n* = 1 UT) out of 10 piglets showed clinical signs of lameness. Furthermore, within the first 5 days after infection, 4 out of 5 piglets of both groups (with or w/o buprenorphine treatment) showed an increased inner body temperature of ≥ 40.2 °C. In the second experiment with a higher infection dose of 2 × 10^8^ CFU, 4 piglets, 2 BT and 2 UT, were recumbent due to high grade lameness on more than one limb (polyarthritis) or showed central nervous disorders and were euthanized 1 dpi. One further UT piglet acutely succumbed to *S.* *suis* disease (Fig. 1B). On the second day following infection, one BT piglet was euthanized due to ongoing moderate disturbed condition with intermittent fever, lameness and reduced appetite (Fig. 1B). Six (*n* = 2 BT, *n* = 4 UT) out of 16 piglets showed signs of low- to high-grade lameness. Elevated body temperature of ≥ 40.2 °C was recorded in 6 out of 8 piglets of both groups (UT and BT). Only 3 out of 10 and 4 out of 16 piglets showed no notable signs of *S.* *suis* disease in the first and second experiment, respectively. The higher infection dose in the second experiment led to higher morbidity and mortality rates in piglets. Piglets with mild clinical signs of disease recovered within 5 dpi in both experiments. However, swollen joints lasted longer in a few piglets. In both experiments BT piglets were more active within half an hour after analgesic injection during the whole treatment period. No negative side effects were observed as a result of buprenorphine treatment. For a comparison of the groups, the sum of the maximal clinical and pain scores in each group were divided by the number of animals to calculate γ and δ, respectively. In the first experiment, UT and BT piglets (each *n* = 5) reached γ values of 7.8 (± 9.93 SD) and 8.0 (± 6.04 SD), respectively. In the second experiment with the higher infection dose, γ values of 13.6 (± 10.78 SD) and 13.8 (± 10.38 SD) were obtained in UT and BT piglets, respectively (*n* = 8 per group, Table 1). In the latter experiment, the mean maximal pain scores δ also reached comparable values in UT and BT piglets with 23.0 (± 23.0 SD) and 21.6 (± 20.6 SD), respectively. In the first experiment with the lower infection dose, UT and BT (*n* = 5) piglets showed δ values of 13.2 (± 20.8 SD) and 8.8 (± 7.3 SD), respectively (see Supplementary Table 3, Additional file 2). The higher value for the UT piglets resulted from the one piglet with central nervous disorder. In summary, significant differences between UT and BT piglets in mortality (Fig. 1A + B); morbidity (Fig. 1C + D) or severe morbidity (Fig. 1E + F) were not recorded. Application of 0.05 mg buprenorphine/kg for 5 days i.m. every 8 h post infectionem did not prevent high clinical and pain scores in affected animals and did not result in substantially lower mean clinical or pain scores.

**Table 1:** Highest clinical score of each *S.* *suis cps*3 infected piglet with or w/o buprenorphine treatment

infection dose: 2 × 10^7^ CFU						infection dose: 2 × 10^8^ CFU					
untreated			buprenorphine-treated			untreated			buprenorphine-treated		
animal number	score	dpi	animal number	score	dpi	animal number	score	dpi	animal number	score	dpi
**1**	4	3	**6**	0	/	**1**	25^†^	1	**9**	5	5
**2**	0	/	**7**	9	1	**2**	17	2	**10**	3	3
**3**	25^a^	2	**8**	4	14	**3**	25^a^	1	**11**	8	1
**4**	7	1	**9**	12	2	**4**	3	2	**12**	17	1
**5**	3	1	**10**	15	1	**5**	2	2	**13**	25^a^	1
						**6**	25^a^	1	**14**	25^a^	1
						**7**	11	3	**15**	2	1
						**8**	1	1	**16**	25^†^	2
γ^b^	**7.8**		γ^b^	**8.0**		γ^b^	**13.6**		γ^b^	**13.8**	

^A^prematurely euthanized piglets due to reaching humane endpoints

^b^γ = Σscore_max_/n_animals_

### Post mortem findings of *S.* *suis cps*3 infected piglets with or w/o analgesic buprenorphine treatment

**Fig. 2:**
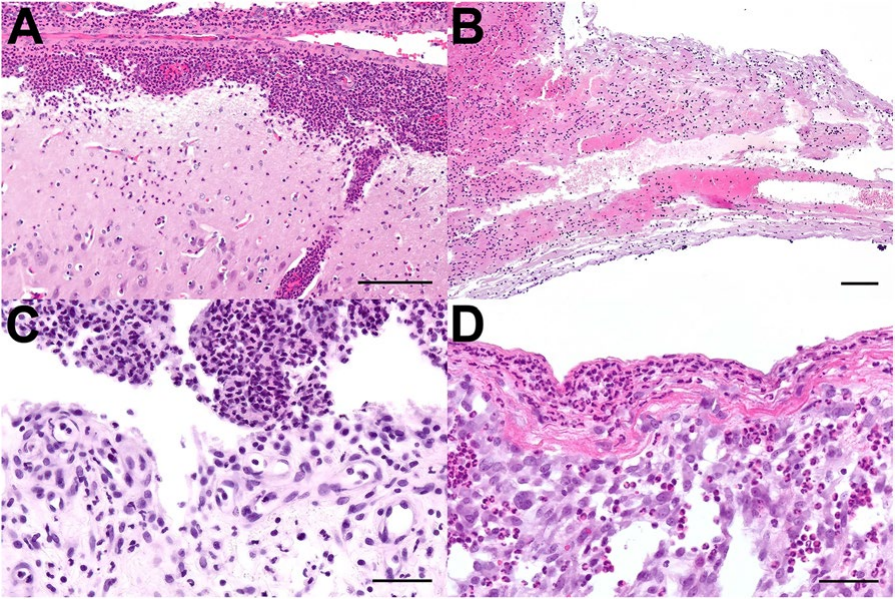
Histopathological findings in piglets experimentally infected with *S. suis cps*3. **A** Acute, suppurative meningoencephalitis in the cerebellar cortex of a BT piglet euthanized 1 dpi. **B** Acute, necrotizing and fibrinosuppurative endocarditis valvularis in the mitral valve of a BT piglet euthanized 1 dpi. **C** Acute, suppurative arthritis in the carpal joint of an UT piglet euthanized 1 dpi. **D** Subacute fibrinosuppurative arthritis with synoviocyte hypertrophy and -hyperplasia in the tarsal joint of a BT piglet euthanized 13 dpi. Hematoxylin–eosin. Bars A, B = 50 µm; C, D = 20 µm

Bacteriological and histopathological investigations of all euthanized piglets were conducted with predefined tissue samples and swabs (*n* = 15 samples per animal) (see Tables 2 and 3). In all mortally ill piglets except one, the challenge strain was isolated from typical samples such as cerebrospinal fluid (CSF) and joint fluid in association with central nervous system disorder and lameness, respectively (Table 2). In three UT and two BT piglets infected with 2 × 10^8^ CFU, the *cps*3 challenge strain was isolated from multiple inner organs, indicating a disseminated infection. These five piglets were euthanized early as they reached predefined humane endpoints. The challenge strain was not detected in inner organs of the piglet euthanized 2 dpi and in the surviving piglets. We applied a scoring system for fibrinosuppurative lesions which we have been using for many years of *S.* *suis* research [14, 28, 29]. This includes calculation of ω as the mean maximal score of each group. UT piglets reached a lower histopathological score of ω = 1.6 compared to BT piglets with ω = 2.5 (see Table 3). Three piglets (one UT and two BT) showed severe fibrinosuppurative meningoencephalitis (Fig. 2A; Table 3). Furthermore, acute fibrinosuppurative endocarditis valvularis was detected in one BT piglet euthanized 1 dpi (Fig. 2B), whereas another UT piglet euthanized 13 dpi had a subacute to chronic endocarditis including granulation tissue. In addition, mild to severe fibrinosuppurative arthritis was detected in 2 piglets of each group (Table 3). Three of these piglets were euthanized 1 dpi due to severe clinical signs in association with acute fibrinosuppurative arthritis (Fig. 2C). In contrast, histopathological findings of the fourth piglet, which survived until the end of the observation period (13 dpi) showed subacute to chronical fibrinosuppurative arthritis including lymphocytes and granulation tissue (Fig. 2D). Except for one piglet, fibrinosuppurative lesions were detected in all prematurely euthanized piglets.

**Table 2:** Reisolation of the *S.* *suis cps*3 challenge strain from piglets after intravenous application of 2 × 10^8^ CFU

buprenorphine	Number of piglets positive for the isolation of the challenge strain in an inner organ^b^, serosa or joint fluid	Number of piglets positive for the isolation of the challenge strain in ≥ 2 organs^b^	Number of piglets in which the *S.* *suis* challenge strain^a^ was isolated from							
			Tonsils^h^	Lung^c^	Serosa^d^	Spleen	Liver	Brain, CSF^e^	Joint fluid^f^	Endocard
untreated	3^ g^/8	3^ g^/8	2/8	1/8	0/8	2/8	1/8	1/8	2/8	1/8
treated	2^ g^/8	2^ g^/8	3/8	2/8	2/8	2/8	2/8	2/8	1/8	2/8

^a^The challenge strain was identified by MP- PCR combined with a serotype specific monoplex PCR

^b^Inner organ refers to lung, spleen, liver, brain, CSF or endocard but not the tonsils

^c^One cranial lobe was investigated

^d^Pleural, peritoneal or pericardial cavity

^e^Cerebrospinal fluid

^f^Punctures of both tarsal and carpal joints were investigated in each animal. In case of lameness additional joint punctures of the respective limb were screened

^g^Reisolation of the challenge strain only in prematurely euthanized piglets

^h^Reisolation of challenge strain from tonsils in one prematurely euthanized and four surviving piglets

**Table 3:** Scoring of fibrinosuppurative lesions of piglets challenged with 2 × 10^8^ CFU of *S.* *suis* strain 16667/3

buprenorphine	piglets without lesions^a^	piglets with lesions in two or more locations^a^	brain			serosae			joint			spleen and liver			lung			heart			
			meningo-encephalitis, chorioiditis			pleuritis or peritonitis^g^ or pericarditis			arthritis			splenitis^b^ or hepatitis			pneumonia			endocarditis			
			5^c^	3^d^	1^e^	4^c^	2^d^	1^e^	4^c^	2^d^	1^e^	4^c^	2^d^	1^e^	4^c^	2^d^	1^e^	4^c^	2^d^	1^e^	ω^f^
untreated	4/8	1^ h^/8	1^i^/8	0/8	1/8	0/8	0/8	0/8	0/8	2/8	0/8	0/8	0/8	0/8	0/8	0/8	1/8	0/8	1/8	0/8	1.6
treated	5/8	2^ h^/8	2^i^/8	0/8	0/8	0/8	0/8	0/8	1/8	1/8	0/8	0/8	0/8	0/8	0/8	0/8	0/8	1/8	0/8	0/8	2.5

^a^Only fibrinosuppurative lesions are considered. Individual single perivascular neutrophils are not counted

^b^Neutrophilic accumulation of the splenic red pulp

^c^Scoring of 4 and 5 indicates moderate to severe diffuse or oligo-/multifocal fibrinosuppurative inflammations

^d^Scoring of 2 and 3 indicates mild focal or multifocal fibrinosuppurative inflammation

^e^Individual single perivascular infiltration with neutrophils received a score of 1

^f^ω = Σscore_max_/n_animals_

^g^Peritoneum is missing from one animal of each group

^h^Fibrinosuppurative lesions in two or more joints

^i^Fibrinosuppurative lesions in several brain areas and the plexus choroideus

### Impact of buprenorphine treatment on the development of antibody and blood leukocyte levels after experimental *S. suis cps*3 infection

**Fig. 3:**
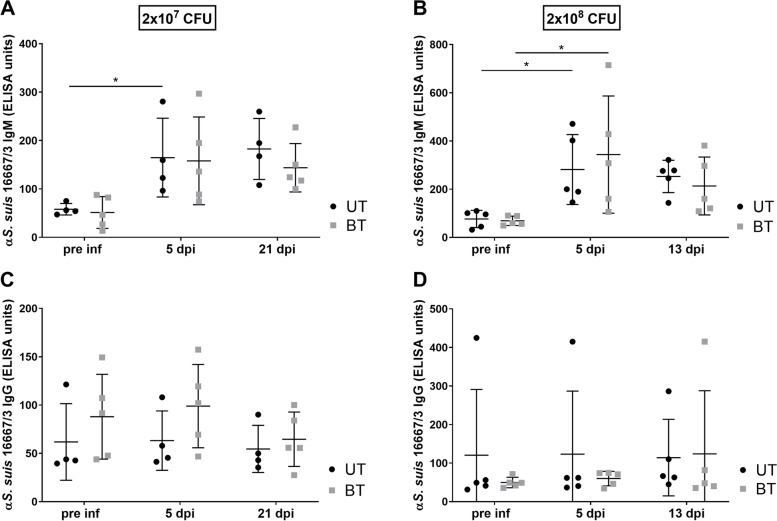
α*S. suis cps*3 serum IgM (**A**, **B**) and IgG (**C**, **D**) levels in piglets with or w/o buprenorphine treatment. Serum was taken from untreated (UT) (●) and buprenorphine-treated (BT) (■) piglets on the indicated time points and the course of α*S.* *suis* 16667/3 IgM (**A** + **B**) and IgG (**C** + **D**) antibodies was determined by ELISA using inactivated, immobilized *S.* *suis cps*3 as antigen. Piglets were intravenously infected with *S.* *suis cps*3 with the infection dose indicated at the top. One group per experiment was treated i.m. with 0.05 mg/kg buprenorphine 0 to 5 days post infection (dpi) every 8 h. Prematurely euthanized piglets were excluded from this analysis. Significant differences in antibody levels between buprenorphine treated and untreated piglets were not recorded. Mean values are indicated by horizontal lines, standard deviations by error bars. Statistical analysis was conducted with the Mann–Whitney-*U*-test (comparison of groups) or one-way ANOVA and subsequently the Dunn’s multiple comparisons test (comparison of time points). Significances are indicated (* *p* < 0.05)

As the immune response of pigs after experimental *S. suis* infection is an important issue in vaccine and pathogenesis studies [15, 30–32], the impact of repeated buprenorphine application on the antibody response and number of blood leukocytes after experimental infection was investigated. Anti (α) *S.* *suis* 16667/3 IgM and IgG antibody levels were determined pre infectionem (pre inf), 5 dpi and at the end of the observation periods 21 dpi or 13 dpi in the first and second experiment, respectively. Piglets prematurely euthanized post infection were not included in this analysis. In the first experiment, a significant increase in α*S. suis* 16667/3 IgM antibodies was determined in the UT group (*n* = 4) with mean ELISA units of 58.1 (± 12.0 SD) pre inf and 164.8 (± 81.4 SD) ELISA units 5 dpi (Fig. 3A). A comparable increase was recorded in the BT group (*n* = 5), though differences were not significant (Fig. 3A). Whereas in the second experiment a significant increase in α*S.* *suis* 16667/3 IgM antibodies was shown for UT (*n* = 5) and BT (*n* = 5) piglets with a mean of 75.4 (± 34.9 SD) and 68.9 (± 19.4 SD) ELISA units, respectively, pre inf to a mean of 281.7 (± 145.0 SD) and 343.5 (± 243.0 SD) ELISA units 5 dpi. Mean ELISA units decreased in both groups from 5 to 13 dpi (Fig. 3B). No significant differences in α*S.* *suis* 16667/3 IgM antibody levels between UT and BT piglets were recorded at any investigated time point. In both experiments no significant change in α*S.* *suis* 16667/3 IgG levels were detected in both groups (Fig. 3C + D). α*S.* *suis* 16667/3 IgG antibodies were not significantly different in UT and BT treated piglets.

In both experiments blood leukocytes increased after experimental infection of pigs with *cps*3 strain 16667/3. In the first experiment, an increase of mean leukocytes from 13.0 G/L (± 4.6 SD; UT *n* = 5) and 14.2 G/L (± 3.5 SD; BT *n* = 5) pre inf to 26.4 G/L (± 14.3 SD; UT) and 23.0 G/L (± 3.5 SD; BT) was detected 2 dpi. Mean leukocytes of the UT group 2 dpi included one piglet which had reached the humane endpoint and was euthanized with blood leukocytes of 50.4 G/L. Blood leukocytes decreased to about 20.0 G/L at 5 dpi in both groups (*n* = 4/UT; *n* = 5/BT) (see Supplementary Fig. 1A, Additional file 3). In the second experiment, blood leukocytes of UT and BT piglets rose from a mean of 14.6 G/L (± 5.1 SD; *n* = 8) and 16.6 G/L (± 4.3 SD; *n* = 8) to a mean 2 dpi of 19.9 G/L (± 4.2 SD; *n* = 5) and 27.7 G/L (± 9.4 SD; *n* = 6), respectively. Mean leukocytes 2 dpi of the BT group included a piglet prematurely euthanized because of reaching humane endpoints with blood leukocytes of 40.0 G/L. One dpi further piglets were prematurely euthanized and showed mean leukocytes of 23.4 G/L (± 13.1 SD; UT *n* = 3) and 17.9 G/L (BT; *n* = 1) (see Supplementary Fig. 1B, Additional file 3). However, mean leukocytes of the UT group 1 dpi included a pig succumbing to acute disease with very low blood leukocytes of 9.3 G/L. No significant differences between UT and BT pigs were determined in both experiments.

### Impact of buprenorphine treatment on bactericidal immunity against *S.* *suis*

The bactericidal assay is used by different groups as an in vitro assay in *S. suis* studies to assess killing of this pathogen in blood, e.g. after vaccination of piglets [14, 29, 33, 34]. Killing of *S. suis* in porcine blood is mediated by opsonizing IgM and IgG antibodies and complement activation on the bacterial surface [11]. Bacterial survival of the *cps*3 *S.* *suis* challenge strain (16667/3) was investigated prior to experimental infection in both experiments. The *S.* *suis cps*3 strain 16,667/3 proliferated in later UT and BT piglets prior to infection with a SF of 117.8 (± 13.50 SD) and 90.6 (± 32.75 SD) in the first experiment as well as 99.2 (± 18.79 SD) and 80.0 (± 36.13 SD) in the second experiment, respectively (see Supplementary Fig. 2A, Additional File 4). Five dpi no differences in survival of the *cps*3 strain were detected between UT and BT piglets, as the strain was efficiently killed (SF = 0.0) in both groups and experiments. Survival of the heterologous *cps*4 and *cps*14 strains in porcine blood was also investigated 5 d after infection with the high dose of 2 × 10^8^ CFU (Supplementary Fig. 2B, Additional file 4). The *cps*4 strain 17434 proliferated in both groups with a mean survival factor (SF) of 46.7 (± 12.93 SD) in the UT group (*n* = 5) and a mean SF of 52.5 (± 8.73 SD) in the BT group (*n* = 5). In contrast, the *S.* *suis cps*14 strain V3117/2 was killed in both groups with a mean SF of 0.09 (± 0.16 SD) and 0.03 (± 0.06 SD) in the UT and BT group, respectively (Supplementary Fig. 2B, Additional file 4). We measured microbial adhesion to hydrocarbons (MATH) of the *S. suis* strains to collect hints for reduced capsule expression. As shown in Supplementary Fig. 3 (Additional file 5), the MATH assay revealed comparable values of *S. suis cps*3 strain 16667/3, *cps*4 strain 17434 and *cps*14 strain V3117/2 and significant differences to the unencapsulated mutant 10*cps*ΔEF for all three strains. This indicates that all three wt strains investigated in the bactericidal assay are encapsulated. In summary, differences in bacterial survival in blood drawn from BT treated and untreated piglets were not recorded.

In the first experiment, we conducted a further bactericidal assay 21 dpi which included a comparison with littermates sampled in the original herd. Whereas *S. suis cps*3 was again efficiently killed in blood drawn from all UT experimentally infected piglets, it showed high proliferation rates in blood of the non-infected littermates (Supplementary Fig. 2C, Additional file 4). However, levels of IgM and IgG binding to immobilized *S. suis cps*3 were not significantly different between *cps*3-infected and non-infected piglets (Supplementary Fig. 2C, Additional file 4). In conclusion, experimental *S. suis cps*3 infection results in immunity leading to efficient killing of *S. suis cps*3 in porcine blood.

### Serum and saliva cortisol levels in pigs infected with *S. suis cps*3 with or w/o buprenorphine treatment

**Fig. 4:**
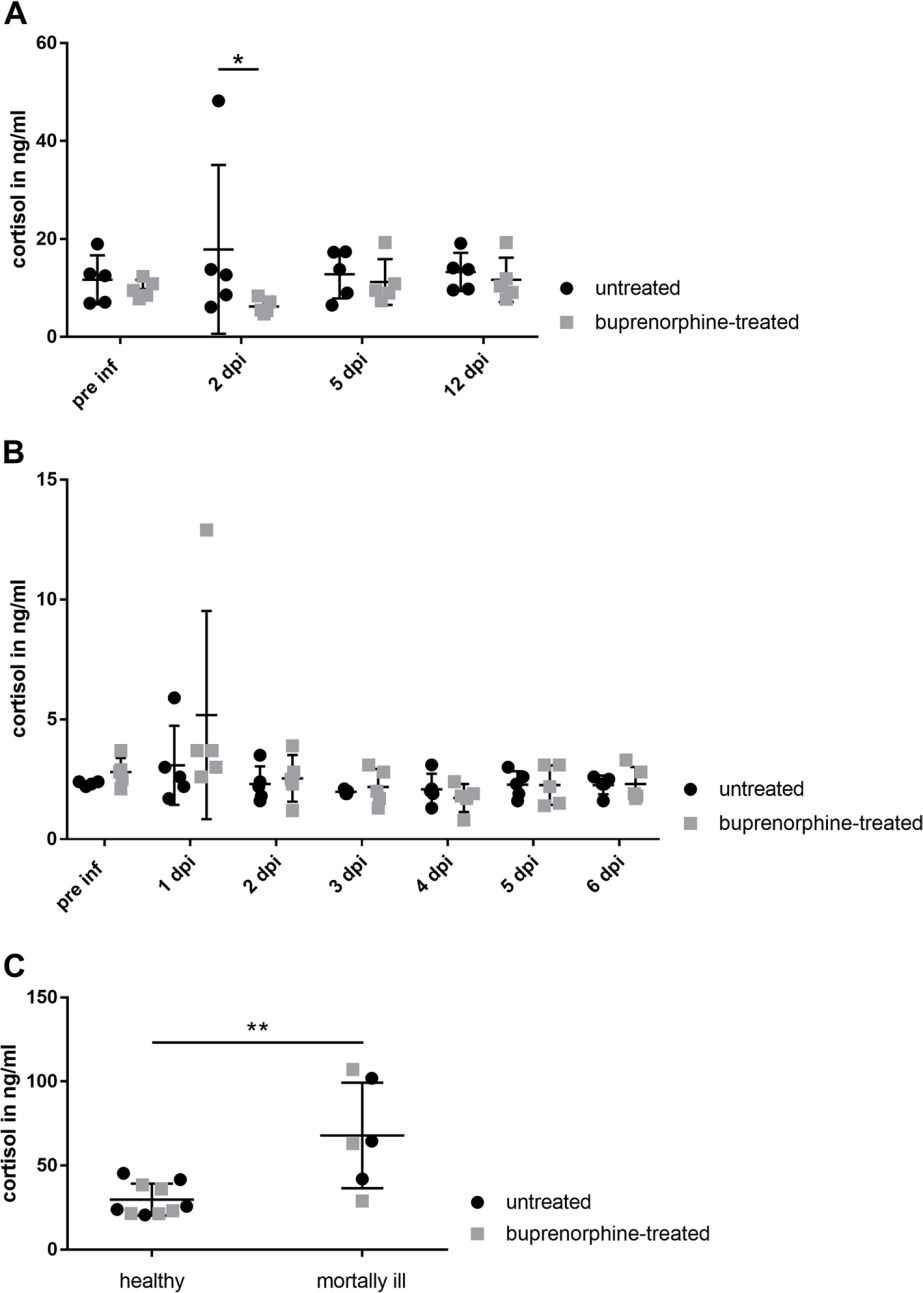
Serum (**A**; **C**) and saliva (**B**) cortisol in *S.* *suis cps*3 infected piglets with or w/o buprenorphine treatment. **A** + **B** Serum and saliva of buprenorphine-treated (*n* = 5) (■) and untreated (*n* = 5) (●) piglets were taken before (pre inf) and at the indicated times after infection with 2 × 10^8^ CFU of *S.* *suis cps*3 strain 16667/3 to determine serum (**A**) and saliva (**B**) cortisol levels. As indicated, one group was treated i.m. with 0.05 mg/kg buprenorphine every 8 h 0 to 5 days post infectionem (dpi). Prematurely euthanized piglets with clinical signs of severe disease were excluded in **A** and **B**. One untreated piglet which showed clinical signs of *S.* *suis* disease had very high serum cortisol level (> 40 ng/ml) 2 dpi (**A**) and additionally high saliva cortisol levels (> 5 ng/ml) 1 dpi (**B**). The buprenorphine-treated piglet with the highest cortisol saliva level (> 10 ng/ml) was morbid on the 1^st^ dpi, but showed prominent recovery thereafter. **C** Comparison of serum cortisol levels of clinically healthy (*n* = 10) and mortally ill (*n* = 6) piglets infected with 2 × 10^8^ CFU *S.* *suis cps*3 and sampled under anaesthesia prior euthanasia. The serum of clinically healthy piglets was collected 13 dpi, whereas in mortally ill piglets, final serum samples were collected within 1–2 dpi after reaching predefined humane endpoints. Mean values are indicated by horizontal lines, standard deviations by error bars. Statistical analysis was conducted with the Mann–Whitney-*U*-test (comparison of groups). Significances are indicated (* *p* < 0.05, ** *p* < 0.01)

Cortisol is a common parameter to measure distress in pigs, as it increases during pain induced stress [35]. We measured serum and saliva cortisol levels as a putative distress marker in piglets experimentally infected with *S.* *suis* to further evaluate buprenorphine treatment as refinement. First, we focused on the piglets that survived until the end of the observation period of 21 (first experiment) or 13 d (second experiment). In the first experiment with an infection dose of 2 × 10^7^ CFU, no substantial differences in blood cortisol levels were found between UT (*n* = 4) and BT (*n* = 5) piglets post *S.* *suis* infection, apart from higher cortisol levels 2 dpi in the BT group with mean cortisol levels of 16.9 ng/ml (± 19.52 SD) compared to the UT group with 10.9 ng/ml (± 3.64 SD) (see Supplementary Fig. 4, Additional file 6). This difference was due to one piglet in the BT group with a serum cortisol level of 51.0 ng/ml. This piglet showed temporally lameness, kyphosis, tremor and anorexia 1 dpi. In the first experiment, cortisol levels pre inf and 21 dpi were generally higher (> 20 ng/ml) compared to the other time points (see Supplementary Fig. 4, Additional file 6), although piglets were clinically healthy. Blood sampling was carried out during anaesthesia at these time points which may explain the results. Accordingly, all serum samples used for the assessment of cortisol levels in the second experiment with an infection dose of 2 × 10^8^ CFU were taken without anaesthesia. No significant differences in cortisol levels were recorded between groups of the second experiment pre inf, 5 dpi and 12 dpi. Mean cortisol levels were below 14 ng/ml at all three time points (Fig. 4A). However, a significantly higher mean cortisol level was determined in the UT group (*n* = 5) 2 dpi with 17.9 ng/ml (± 17.2 SD) compared to 6.2 ng/ml (± 1.5 SD) in the BT group (*n* = 5) (Fig. 4A). In the UT group one piglet obtained a very high serum cortisol level of 48.2 ng/ml 2 dpi in association with lameness, kyphosis and local tremor.

In the second experiment with the higher infection dose we additionally collected saliva every day for 6 days following infection to measure cortisol levels in saliva. We excluded mortally ill piglets due to the obligation to euthanize these piglets already 1 and 2 dpi. Significant differences in saliva cortisol levels were not recorded with or w/o buprenorphine treatment (Fig. 4B). Of note, one piglet in each group showed elevated saliva cortisol levels with values above 5 ng/ml 1 dpi. These piglets showed clinical signs of disease such as elevated inner body temperature (≥ 40.2 °C), reduced behavior and high-grade lameness. Additionally, one of these piglets showed anorexia, kyphosis and local tremor. All other cortisol values were below 5 ng/ml and thus comparable to the values pre inf. These results suggest that piglets do not go through severe distress post i.v. *S.* *suis* infection as long as they are clinically unobtrusive.

To evaluate cortisol as a distress marker in piglets experimentally infected with *S.* *suis*, serum cortisol levels of healthy piglets drawn under anaesthesia at the end of the observation period of 13 dpi were compared to levels in serum samples drawn from anaesthetized, mortally ill piglets prior euthanasia 1 dpi and 2 dpi. Highly significant differences between the aforementioned groups were found with mean cortisol levels of 29.8 ng/ml (± 9.52 SD) in healthy (*n* = 10) and 68.0 ng/ml (± 29.54 SD) in mortally ill (*n* = 6) piglets (Fig. 4C). These results show that cortisol is a meaningful distress marker in piglets infected experimentally with *S. suis* and confirm the importance of humane endpoints and a close clinical monitoring as refinement. However, buprenorphine treatment did not prevent high serum cortisol levels in diseased piglets. Furthermore, it does not indicate differences of saliva cortisol levels in clinically unobtrusive piglets infected with *S.* *suis cps*3.

## Discussion

Experimental infections of pigs and mice with different *S.* *suis* strains have revealed that there are substantial differences in virulence between strains of different *cps* but also within certain *cps* such as *cps*2 [36, 37]. This includes *cps*2 strains which are avirulent even in germfree piglets [36] and highly virulent *cps*9 strains causing death within hours after application [29]. Here, we describe experimental induction of suppurative meningoencephalitis and arthritis, the most important *S. suis* associated pathologies in the field, in 6-week-old piglets through application of a *cps*3 strain for the first time. Furthermore, the *cps*3 strain induced acute to subacute and chronical endocarditis (see Fig. 2 and Table 3). Though endocarditis has been observed after experimental infection with other *S. suis cps* [38], the detection of a necrotizing and fibrinosuppurative endocarditis valvularis already after 24 h after experimental infection is remarkable (Fig. 2B). As the challenge strain was isolated from the brain, CSF, joints and heart valve of these animals (Table 2) the third Henle-Koch postulate was proven for this important *cps* in the natural host. Intravenous application of 2 × 10^7^ CFU resulted in high morbidity (Fig. 1C), but low mortality rates (Fig. 1A). Whereas the higher infection dose of 2 × 10^8^ CFU led to high morbidity (Fig. 1D), moderate mortality rates (Fig. 1B) and typical suppurative or fibrinosuppurative lesions (Fig. 2; Table 3) in 6-week-old pigs.

*S. suis* is an invasive porcine pathogen causing severe diseases such as meningitis, arthritis and septicemia [39]. Therefore, we evaluated the treatment of experimentally infected piglets with buprenorphine, an opioid analgesic, as a refinement strategy in experimental *S.* *suis* pig infections to reduce pain and distress of animals. For this purpose, data on mortality, morbidity and clinical as well as pain scores were recorded and compared. In previous studies, buprenorphine treatment was able to reduce pain and lameness in pigs [26]. Although numerous piglets suffered from lameness after i.v. *cps*3 infection, no substantial differences regarding the aforementioned investigated parameters were recorded between UT and BT pigs. Although the number of piglets in this trial is limited, our data indicate that the selected buprenorphine application is not sufficient to prevent severe pain and clinical signs in pigs infected intravenously with *S. suis cps*3.

One may argue that the applied dose of 0.05 mg buprenorphine/kg was too low to prevent high clinical and pain scores in diseased piglets, since it was shown that a dose of 0.05 mg/kg was not able to reduce surgical pain in mice [40]. However, the applied and published doses of buprenorphine in swine range from 0.005 to 0.1 mg/kg [41]. As buprenorphine can go along with negative side effects like respiratory depression and sedation [19], the dose should not be chosen too high. Previous publications regarding buprenorphine treatment in pigs reported no negative side effects with an applied dose of 0.04 mg/kg [26, 27]. Based on the doses used in published studies and recommended through GV- Solas for pain management in laboratory animals [19], the applied dose of 0.05 mg/kg was considered to be adequate. Apart from the dose there may be further reasons for the insufficient effect of buprenorphine treatment on pain reduction. Of note, buprenorphine treatment did not lead to restoration of the symmetric gait of lame pigs as determined with pressure mats in a previous study [27]. As post mortem examinations showed that most of the piglets suffered from inflammatory conditions resulting in lameness, it was suspected that buprenorphine was not able to reduce pain induced through inflammatory disease, which was still investigated in a previous study [41]. We assume that in our study the effects of buprenorphine on clinics and cortisol levels were very limited, as *S.* *suis* typically induces a high-grade of inflammation in association with lameness in pigs. Possibly, drugs with a strong anti-inflammatory effect are more likely to result in a detectable reduction of the clinical and pain score. However, as inflammation is an important read out parameter in *S.* *suis* studies, such a refinement strategy is not in accordance with the scientific objective of most experimental *S. suis* studies.

Although, morbidity and clinical signs were similar between both groups, BT piglets were notably more active within 30 min after injection compared to UT piglets. Especially after anaesthesia for experimental infection, BT piglets were awake and active earlier compared to UT. This increase in active movement after buprenorphine treatment of pigs is in agreement with other studies [26, 27]. Within this study, a few BT piglets showed clinical signs of high fever and severe lameness. However, these piglets did not reach humane endpoints, since they were still active and showed appetite. Though our data on the clinics (Fig. 1) does not indicate different disease progression under buprenorphine treatment, we cannot rule out that buprenorphine treatment may have led to delayed euthanasia and prolonged the course of disease in single cases.

In our established *cps*3 infection model central nervous disorder and lameness were common clinical findings. This was associated with acute suppurative meningoencephalitis and arthritis in 5 out of 6 prematurely euthanized piglets, respectively, as well as subacute to chronical arthritis in one surviving piglet. Of note, the mean pathohistological score ω was substantially higher in BT than in UT piglets though morbidity and mortality were overall very much alike between the two groups. One might speculate that BT treated piglets reached higher pathohistological scores because buprenorphine treatment disguised early clinical signs in the onset of disease leading to delayed euthanasia in piglets developing (fibrino-)suppurative lesions. However, BT treated piglets reaching humane endpoints were euthanized within the first two days following experimental infection (Fig. 1) and typical clinical signs such as lameness were not prevented by buprenorphine treatment. Overall, the results of the clinical monitoring suggest that the applied buprenorphine treatment has very little influence on the clinical manifestation of experimentally induced *S. suis* disease.

Interestingly, piglets which showed clinical signs of *S.* *suis* infection and did not reach humane endpoints, recovered within 5 d after infection independent from buprenorphine treatment. This result is consistent with data from the bactericidal assay of the *cps*3 challenge strain, since the strain proliferated in porcine blood drawn before infection and was killed completely in blood taken 5 d after infection. Both experiments showed increasing levels of IgM antibodies against the *cps*3 challenge strain following experimental infection (already 5 dpi). Likewise, this was shown in mice after sublethal infection with a *S. suis cps*3 strain, leading to a significant increase in IgM antibodies against *cps*3 [42]. The aforementioned study further showed that the increase in IgM antibodies is independent of the subcapsular domain of the *cps*3 strain. Whether the fast recovery of the infected and diseased piglets, as well as the killing of the *cps*3 strain in porcine blood ex vivo 5 dpi is mediated by the increased IgM levels needs further investigation.

As shown, opioid analgesics are able to modulate the innate and acquired immune response [19, 24, 25]. However, this study indicates that buprenorphine treatment has no impact on the antibody-mediated killing of the *S.* *suis* challenge strain in porcine blood, as well as on levels of blood leukocytes and specific IgM and IgG antibodies in pigs. These results go along with the fact, that buprenorphine has less immunomodulatory effects as a partial µ-agonist compared to e.g. fentanyl or morphine as pure µ-agonists [22]. Buprenorphine is not activating the hypothalamic–pituitary–adrenal-axis. Therefore, buprenorphine does not induce immunomodulatory glucocorticoids [24]. After ocular infection of guinea pigs with a virulent *Shigella flexneri* strain, buprenorphine treatment did not influence the IgG and IgA antibody response [43]. Accordingly, we show that buprenorphine treatment of *S.* *suis* infected pigs is neither associated with different bacterial survival patterns in porcine blood nor with altered serum levels of IgM and IgG antibodies binding to the bacterial surface of *S. suis cps*3. This suggests that the immunomodulatory effect of the selected buprenorphine application is neglectable with regard to the humoral immune response following *S. suis cps*3 infection.

Scientists working with animals are obliged to implement refinement strategies in experiments to reduce distress. However, these are often poorly described in experimental *S.* *suis* studies and have not been evaluated, though differences between laboratories may have a substantial impact on animal welfare and also on the scientific output. In the described study, we not only evaluated buprenorphine as refinement strategy, but also introduced measurements of cortisol levels as a distress marker in *S suis* research. In our opinion, it is necessary to standardize refinement strategies between different laboratories. For this, it is very important to include objective read out parameters of distress such as serum and saliva levels of cortisol. Importantly, cortisol levels in experimentally infected piglets were significantly associated with clinical signs of disease (Fig. 4C).

As increased cortisol levels in pigs typically go along with pain caused distress in pigs [23, 44], serum cortisol levels in *S.* *suis* infected piglets with or w/o buprenorphine treatment were determined. We showed that serum cortisol levels were significantly higher in mortally ill piglets suffering from *S.* *suis* disease compared to healthy piglets. This confirms that serum cortisol levels relate with *S.* *suis* disease and can be used as distress marker. In this study buprenorphine treatment did not prevent high serum cortisol levels in *S.* *suis* infected pigs with severe clinical signs (Fig. 4C). This indicates that humane endpoints and a close clinical monitoring should remain obligatory refinement strategies independent of buprenorphine application to reduce distress in piglets.

The UT piglets were intentionally not treated with a placebo in this study because such treatment would not be performed in any experimental *S. suis* study. As a consequence, we cannot rule out that comparison with a placebo-treated group would have resulted in more prominent differences in cortisol levels between both groups. We consider i.m. injection of buprenorphine every 8 h post infection to be stressful for the animals, at least to a low degree. As buprenorphine application did not prevent high clinical and pain scores, the benefit of it in experimental *S.* *suis* infections remains questionable in our opinion, though differences in serum cortisol concentration were recorded 2 dpi in the 2^nd^ experiment.

## Conclusion

Different pathologies, e.g. suppurative meningoencephalitis, arthritis and endocarditis associated with typical clinical signs of disease can be induced through i.v. application of 2 × 10^8^ CFU of *S.* *suis cps*3 strain 16667/3 in conventional piglets. In the established *cps*3 challenge model, buprenorphine treatment (0.05 mg/kg every 8 h) does not prevent high clinical and pain scores in single piglets with severe disease limiting its value as putative refinement. Results suggest that buprenorphine treatment has no influence on the course of specific serum IgM and IgG levels in these experimentally infected piglets. Serum cortisol levels are a meaningful marker of distress in piglets experimentally infected with *S.* *suis*, since levels were significantly higher in mortally ill piglets compared to healthy ones. Furthermore, our study suggests that in experimentally infected but clinically unobtrusive piglets, serum and saliva cortisol levels remain low and are not influenced by buprenorphine application. Overall, close clinical monitoring in combination with well-defined humane endpoints remains the main refinement strategy to limit distress and pain in *S.* *suis* experimentally infected piglets.

## Materials and methods

### Bacterial strains and growth conditions

*S. suis* strain 16667/3 (*cps3*^+^, *sly*^+^, *mrp*^+^) of ST 1187 was originally isolated from the central nervous system of a diseased pig in Germany in 2016. *S. suis* strain V3117/2 (*cps14*^+^, *sly*^+^, *mrp*^+^, epf*) of ST1 was isolated from the brain of a pig as described before [45]. The *S.* *suis* strain 17434 (*cps4*^+^, *sly*^+^, *mrp*^+^) of ST94 was isolated from the CSF of a pig in Germany in 2017. *S. suis* strain 10*cps*ΔEF, kindly provided by Hilde Smith (DLO-Institute for Animal Science and Health, Lelystad, The Netherlands), is an unencapsulated mutant derived through insertion of a spectinomycin resistance cassette in *cps*E and *cps*F of the capsule biosynthesis operon of *S. suis cps*2 wt strain 10 [46]. Bacterial strains were cultured on Columbia agar plates containing 5% sheep blood and if appropriate *Staphylococcus*/*Streptococcus* selective Supplement (SR0070E, Oxoid, Germany) was added. Furthermore, bacteria were cultured in Bacto™ Todd Hewitt broth (THB) and Bacto™ Tryptic Soy broth (TSB).

### Virulence-associated gene profiling and multi locus sequence typing (MLST)

The virulence-associated genes of the aforementioned *S. suis cps*3 and *cps*4 strains were determined through MP-PCR [5]. Capsular polysaccharide synthesis genes for the detection of serotypes 3 and 4 (*cps*3 and 4) were detected through monoplex PCR as described [47]. MLST was conducted as published previously [45].

### Animal experiment

In this study, all pigs were treated in accordance with the principles outlined in the EU Directive 2010/63/EU, the European Convention for the Protection of Vertebrate Animals Used for Experimental and other Scientific Purposes and the German Animal Protection Law (Tierschutzgesetz). The animal experiment (permit no. TVV58/20) was permitted by the Landesdirektion Sachsen. The animal experiment included two studies with different applied infection doses. All piglets (BT and UT) of one experiment were kept in the same stable. The person (CGB) who made the final decision on early euthanasia did not know which group (BT or UT) an animal belonged to (partially blinded experiment). Piglets included in the study originated from a conventional farm with several circulating *S. suis cps* including *cps*1, *cps*1/2, *cps*2, *cps*7 and *cps*9 as determined by genotyping of numerous tonsil isolates using (MP)-PCR [5] followed by *cpsK* sequencing to differentiate between *cps*1 and *cps*14 and between *cps*2 and *cps*1/2 [48]. *S. suis* strains isolated from tonsil swabs taken from piglets before experimental infection and further tonsil isolates (*n* = 105) from the same herd were screened by a *cps*3 monoplex PCR as described before [47]. The *cps3* gene was not detected in any of the tonsil isolates. However, this does not completely exclude that the piglets were colonized with *cps*3. As buprenorphine had not been evaluated in experimental *S. suis* studies, it was difficult to give a reasonable estimate for p(x > y) to calculate the sample size for the comparative analysis of animals in clinical and pain scores as well as cortisol levels for the Wilcoxon Mann–Whitney U-test comparison. Accordingly, the 1st experiment with the lower infectious dose was conducted as an orientation study. The sample size of the 2^nd^ experiment with an infectious dose of 2 × 10^8^ CFU was calculated with the following settings: p(x > y) = 0.92; alpha 0.05; power 0.8. These settings relied on the hypothesis that i.v. infection with *S. suis cps*3 should result in very high clinical and pain scores as well as cortisol levels in nearly all untreated piglets (e.g. a clinical score of 25, see Table 1) and that buprenorphine treatment is very sufficient to prevent maximal clinical and pain scores as well as cortisol levels in clinically diseased piglets. The piglets were taken from two (first experiment) or five (second experiment) litters of German Landrace sows crossed with Pietrain. After weaning with four weeks of age, piglets were transported to the experimental facilities and allocated into the UT and BT group applying a litter- and gender-matched design. The single animal represents an experimental unit. The experiment started following an acclimatization period of one week. Stables were enriched with cotton ropes, different plastic toys and chains. After the acclimatization period pigs were infected intravenously with *S.* *suis cps*3 strain 16667/3 in the 6^th^ week of life. The experimental infection was conducted under anaesthesia induced through i.m. application of 2 mg/kg azaperon (Stresnil®, Elanco, Germany) and 10 mg/kg ketamine (Ursotamin®, Serumwerke Bernburg; Germany). In the first experiment, piglets (*n* = 5/group) were experimentally infected with 2 × 10^7^ CFU/1.5 ml/animal. In the second experiment the infection dose was increased to 2 × 10^8^ CFU/1.5 ml per piglet (*n* = 8/group). In both trials, one group was treated i.m. with 0.05 mg/kg buprenorphine (Buprenovet® Multidose; 0.3 mg/ml, Elanco, Germany) every 8 h from the day of infection until 5 dpi. A first dose of 0.01 mg/kg buprenorphine was applied during anaesthesia induced for experimental infection. This first dose was lower compared to subsequent treatments to minimize respiratory depression. Buprenorphine doses were based on recommendations of the GV-Solas for pain treatment in laboratory pigs [19]. Blood from the vena cava cranialis was taken prior to infection, 2 dpi and 5 dpi and during final aesthesia. In the second trial, blood was additionally taken one day prior to final necropsy. Further, saliva of pigs was collected daily starting on the day prior to infection and lasting until 6 dpi in the second experiment. Experimentally infected piglets were fed thrice daily with commercial rearing food and had ad libitum access to water. Furthermore, piglets were clinically monitored every 8 h following a standardized protocol. A clinical score with a maximum of 25 (see Supplementary Table 1, Additional file 1) and a pain score with a maximum of 50 (see Supplementary Table 2, Additional file 1) was determined for each piglet at every control. To record all clinical signs of arthritis, not only lameness, but also signs of inflammation like redness and swelling of joints were scored. The pain score included typical signs of pain in pigs suffering from *S.* *suis* disease and was established for the first time in this experimental infection. The maximal pain scores reached in each group were divided by the number of animals and defined as δ. A pig was defined as morbid if the inner body temperature obtained values ≥ 40.2 °C and/or developed clinical signs of severe *S.* *suis* disease. Piglets were euthanized due to animal welfare reasons if they showed clinical signs of severe *S. suis* disease like polyarthritis, recumbency and central nervous disorder or reached a clinical score ≥ 25. Humane endpoints were also reached, when piglets suffered from ongoing moderate disturbed condition (clinical score from 8–24 over 24 h). For euthanasia, piglets were deeply anaesthetized as described above and euthanized through i.v. application of 3 ml T61 (MSD Tiergesundheit, Germany) via the ear vein. After euthanasia, all challenged piglets underwent the same necropsy protocol to collect the following samples for histological (h) and semi-quantitative bacteriological (b) investigations as described previously [30, 49]: CSF (b); brain (b, h); tarsal and carpal joints (b, h), peritoneal, pleural and pericardial swabs (b), peritoneum and pleura (h); cranial lobe of the left lung (b, h); liver (b, h); spleen (b, h); mitral valve (b, h) and tonsil (b, h). The histological screenings were scored as described [28].

### Bactericidal assay

Venous blood was taken prior to infection, 5 dpi and 13 dpi to perform bactericidal assays which were conducted essentially as described previously [33]. Briefly, 500 µl of heparinized blood (16 I.U. heparin/ml) was infected with 6 × 10^5^ CFU of *S. suis cps*3 strain 16667/3 or 3 × 10^6^ CFU of *S. suis cps*4 strain 17434 and *cps*14 strain V3117/2 for two hours at 37 °C on a rotator. Streptococci for blood infection were grown exponentially in THB to an OD_600_ = 0.5–0.6 and stored at -80 °C until the bactericidal assay. Bacterial growth was determined by plating serial dilutions at time point zero and after two hours. The SF for each sample was calculated through division of these two values.

### Microbial adhesion to hydrocarbons (MATH)

We conducted the MATH assay as described previously by Öhlmann et al. [50] to compare hydrophobicity of the *S. suis* strains investigated in the bactericidal assay with the hydrophobicity of *S. suis cps*2 strain 10 and its unencapsulated isogenic mutant 10*cps*ΔEF.

### Detection of specific α*S. suis* 16667/3 IgM and IgG antibodies

Antibody levels in pig sera were determined prior to infection, 5dpi and at final euthanasia. ELISAs for detection of serum IgM and IgG antibodies binding to immobilized, formaldehyde-inactivated (0.2%) whole bacteria of *S. suis cps*3 strain 16667/3 were conducted as described previously for other *S. suis* strains [45]. Convalescent sera from piglets experimentally infected with *cps*3 strain 16667/3 served as standard serum (piglet no. 954) and positive control (piglet no. 800). These sera were taken three weeks after infection. Serum from colostrum-deprived piglets served as negative control.

### Measurement of blood leukocytes

To determine blood leukocytes in pigs, EDTA blood (Sartstedt, Nürmbrecht, Germany) was drawn prior to infection, 2 dpi and 5 dpi in the first experiment. In addition to the aforementioned time points blood was taken at final euthanasia of pigs in the second experiment. Blood leukocytes were differentiated using the hematology system ADVIA 120 (Siemens Healthineers, Germany) as described previously [51].

### Saliva sampling

Saliva of piglets was collected using round cotton swabs (Covetrus DE GmbH, Düsseldorf, Germany). Prior infection, piglets were trained to chew on swabs using swabs soaked in apple juice. For saliva collection, piglets had to chew on two swabs, one after the other, until they were moist. After every saliva sampling, pigs were rewarded with apple juice-soaked swabs. Due to the circadian rhythm of cortisol [52, 53], saliva was collected daily at noon before feeding until 6 dpi. After sampling, both swabs were placed into a 5 ml syringe, which was subsequently put into a 15 ml falcon tube and closed with parafilm. Tubes were centrifuged for 20 min with 1685 g at room temperature. Saliva was stored at -20 °C until cortisol measurements.

### Determination of serum and saliva cortisol levels

To investigate serum cortisol levels in the first experiment, blood was taken prior to infection, 2 dpi, 5 dpi and at final euthanasia (21 dpi) of pigs. Samples prior to infection and at final euthanasia were taken under anaesthesia in the first experiment with the lower infection dose. Due to the obtained results (see Supplementary Fig. 4, Additional file 6) and published data on the influence of anesthetics on cortisol levels in pigs [54], this was changed in the second experiment: Blood samples were taken from awake piglets one day before infection, 2 dpi, 5 dpi and one day prior to final euthanasia in the case of surviving piglets (12 dpi). Further blood samples were drawn under anaesthesia before final euthanasia of mortally ill piglets (1 dpi and 2 dpi) and surviving piglets (13 dpi). Due to the circadian rhythm of cortisol with higher levels in the morning and noon [52], the time point of sampling is very important. In the second experiment with the higher infection dose, blood was always drawn in the morning and saliva in the noon. However, this was not consistently implemented in the first experiment with the lower infection dose. Because of this and the described differences in anaesthesia, results of cortisol measurement in the first experiment are considered preliminary data and only shown in the Additional file 6. Cortisol levels in saliva and serum were determined by radioimmunoassay as described previously [55].

### Statistical analysis

Statistical analysis was conducted using GraphPad Prism 7.05 (Graph Pad Software, California, USA). Differences between the UT and BT group were analysed using the Mann–Whitney-*U* test. For comparison of two or more different time points within one group one- way analysis of variance (ANOVA) with a subsequent Dunn´s multiple comparisons test was applied. Data shown in the Kaplan–Meier diagrams were analysed using the log rank test. Means and standard deviations of the results are shown. Probabilities lower than 0.05 were considered significant (* *p* < 0.05, ** *p* < 0.01, *** *p* < 0.001, **** *p* < 0.0001).

## Supplementary Information

**Additional file 1: Supplementary Table 1.** Scoring of clinical signs in pigs. **Supplementary Table 2.** Scoring of pain in pigs.

**Additional file 2: Supplementary Table 3.** Highest pain score of each S. *suis* cps3 infected piglets w or w/o buprenorphine treatment.

**Additional file 3: Supplementary Fig. 1.** Course of blood leukocytes in piglets w or w/o buprenorphine treatment after S. *suis* cps3 infection. EDTA blood was taken before (pre inf) and after experimental infection with 2x10^7^ CFU (A) or 2x10^8^ CFU (B) of S. *suis* *cps*3 strain 16667/3. One group was treated i.m. with buprenorphine 0 to 5 days post infection (dpi). Unfilled symbols (○ , □) indicate piglets prematurely euthanized due to reaching humane endpoints. Leukocytes increased after infection in all (A) and 14 of 15 (B) piglets, respectively, and no significant differences between buprenorphine-treated and untreated piglets were determined. Statistical analysis was conducted with the Mann-Whitney-U-test (comparison of groups).

**Additional file 4: Supplementary Fig. 2.** Survival of *S. suis* strains of the indicated cps in blood of BT and UT piglets (A and B) and survival of *S. suis* *cps*3 as well as specific serum IgM and IgG antibody levels in *cps*3 infected and non-infected piglets (C).

**Additional file 5: Supplementary Fig. 3.** Microbial adhesion of S. *suis* *cps*3, *cps*4 and *cps*14 to hydrocarbons.

**Additional file 6: Supplementary Fig. 4.** Serum cortisol in piglets infected with 2x10^7^ CFU S. *suis* *cps*3 w or w/o buprenorphine treatment.

## Data Availability

The datasets supporting the conclusions of this article are included within the article and its additional files. The datasets analyzed during the current study are available from the corresponding author on reasonable request.

## Abbreviations

α: Anti
BT: Buprenorphine-treated
*cps*: Serotype
CSF: Cerebrospinal fluid
i.m.: Intramuscular
i.v.: Intravenous
MLST: Multi locus sequence typing
dpi: Days post infectionem
pre inf: Pre infectionem
SF: Survival factor
*S. suis*: Streptococcus suis
ST: Sequence type
UT: Untreated
