# Supplementary material for: A new S. suis serotype 3 infection model in pigs: lack of effect of buprenorphine treatment to reduce distress
Source: BMC Vet Res. 2022 Dec 12;18:435. doi: 10.1186/s12917-022-03532-w (PMC9743652; doi:10.1186/s12917-022-03532-w)
Supplement: Supplementary file 1 — Additional file 1: Supplementary Table 1. Scoring of clinical signs in pigs. Supplementary Table 2. Scoring of pain in pigs. [file 12917_2022_3532_MOESM1_ESM.docx]

| **Supplementary Table 1.** Scoring of clinical signs in pigs | | | | | | | | | | |
| --- | --- | --- | --- | --- | --- | --- | --- | --- | --- | --- |
| parameter | score | | | | | | | | | |
|  | 0 | 1 | 2 | 3 | 4 | 5 | 8 | 10 | 25 |  |
| body temperature | < 40°C | 40.0 - 40.2°C | 40.3 - 40.5°C | > 40.5°C |  |  |  |  |  |  |
| feed intake | good |  | moderate |  |  | ceased |  |  |  |  |
| lameness; signs of inflammation (redness, swelling) | no lameness or redness and swelling on joints | low-grade lameness; and/or low-grade redness and swelling on one joint |  | high-grade lameness; and or middle to high-grade redness and swelling on one joint |  |  |  |  | recumbency, polyarthritis; and/ or middle to high-grade redness and swelling on several joints^b^ |  |
| behavior | fresh | damped |  |  |  |  |  | listless | central nervous system disorder^a^ |  |
| respiratory signs | costo-abdominal |  | forced abdominal breathing |  |  |  | cyanosis (ears) |  |  |  |
| special signs | species-typical |  | kyphosis | vomiting | local tremor |  | generalized tremor |  |  |  |

**Additional File 1.** Clinical and pain scoring of pigs.

^a^ tetanic spasm, opisthotonus, convulsions

^b^ associated with reduced general condition

| **Supplementary Table 2.** Scoring of pain in pigs | | | | | | | | | | |
| --- | --- | --- | --- | --- | --- | --- | --- | --- | --- | --- |
| parameter |  | score | | | | | | | | |
|  | 0 | 1 | 2 | 4 | 5 | 8 | 10 | 20 | 50 |  |
| feed intake | good | moderate |  |  | ceased |  |  |  |  |  |
| lameness | no |  | low grade |  | High-grade |  |  |  | recumbency, polyarthritis |  |
| movement time | moving with others the whole observation period |  | stands up later/ lays down earlier than others, runs with others for the rest of observation period |  | stands up, runs with others for a very short time |  |  |  |  |  |
| get- and scare up | stands up by his own |  | stands up after scaring up |  |  |  |  |  |  |  |
| pain vocalisation | none |  |  |  | intensive, but temporary |  | intensive and enduring |  |  |  |
| behavior | fresh | damped |  |  | listless |  |  | moderate^a^ central nervous disorder | severe^b^ central nervous system disorder^a^ |  |
| tremor | none |  |  | local tremor |  | enduring, generalized tremor |  |  |  |  |
| kyphosis | species typical |  | temporary^c^ |  | enduring^d^ |  |  |  |  |  |

^a^ opisthotonus, but active movement

^b^ tetanic spasm, opisthotonus, convulsions

^c^ for one observation time

^d^ ≥2 observation times
