# Supplementary material for: A new S. suis serotype 3 infection model in pigs: lack of effect of buprenorphine treatment to reduce distress
Source: BMC Vet Res. 2022 Dec 12;18:435. doi: 10.1186/s12917-022-03532-w (PMC9743652; doi:10.1186/s12917-022-03532-w)
Supplement: Supplementary file 2 — Additional file 2: Supplementary Table 3. Highest pain score of each S. suis cps3 infected piglets w or w/o buprenorphine treatment. [file 12917_2022_3532_MOESM2_ESM.docx]

**Additional File 2.** Highest reached pain scores of piglets.

| **Supplementary Table 3.** Highest pain score of each *S. suis* *cps*3 infected piglets w or w/o buprenorphine treatment | | | | | | | | | | | | | | |
| --- | --- | --- | --- | --- | --- | --- | --- | --- | --- | --- | --- | --- | --- | --- |
| **infection dose: 2x10^7^ CFU** | | | | | | |  | **infection dose: 2x10^8^ CFU** | | | | | | |
| untreated | | |  | buprenorphine-treated | | |  | untreated | | |  | buprenorphine-treated | | |
| animal number | score | dpi |  | animal number | score | dpi |  | animal number | score | dpi |  | animal number | score | dpi |
| **1** | 3 | 3 |  | **6** | 0 | / |  | **1** | 50^†^ | 1 |  | **9** | 2 | 5 |
| **2** | 0 | / |  | **7** | 10 | 1 |  | **2** | 12 | 1 |  | **10** | 0 | / |
| **3** | 50^†^ | 2 |  | **8** | 3 | 4 |  | **3** | 50^†^ | 1 |  | **11** | 28 | 1 |
| **4** | 9 | 1 |  | **9** | 13 | 2 |  | **4** | 2 | 2 |  | **12** | 17 | 1 |
| **5** | 4 | 1 |  | **10** | 18 | 1 |  | **5** | 2 | 2 |  | **13** | 50^†^ | 1 |
|  |  |  |  |  |  |  |  | **6** | 50^†^ | 1 |  | **14** | 50^†^ | 1 |
|  |  |  |  |  |  |  |  | **7** | 17 | 1 |  | **15** | 1 | 1 |
|  |  |  |  |  |  |  |  | **8** | 1 | 1 |  | **16** | 25^†^ | 2 |
| **δ*** | **13.2** |  |  | **δ*** | **8.8** |  |  | **δ*** | **23.0** |  |  | **δ*** | **21.6** |  |

† prematurely euthanized piglets due to reaching human endpoints

* δ = Σscore_max_/n_animals_
