# Supplementary material for: A new S. suis serotype 3 infection model in pigs: lack of effect of buprenorphine treatment to reduce distress
Source: BMC Vet Res. 2022 Dec 12;18:435. doi: 10.1186/s12917-022-03532-w (PMC9743652; doi:10.1186/s12917-022-03532-w)
Supplement: Supplementary file 3 — Additional file 3: Supplementary Fig. 1. Course of blood leukocytes in piglets w or w/o buprenorphine treatment after S. suis cps3 infection. EDTA blood was taken before (pre inf) and after experimental infection with 2x10^7^ CFU (A) or 2x10^8^ CFU (B) of S. suis cps3 strain 16667/3. One group was treated i.m. with buprenorphine 0 to 5 days post infection (dpi). Unfilled symbols (○ , □) indicate piglets prematurely euthanized due to reaching humane endpoints. Leukocytes increased after infection in all (A) and 14 of 15 (B) piglets, respectively, and no significant differences between buprenorphine-treated and untreated piglets were determined. Statistical analysis was conducted with the Mann-Whitney-U-test (comparison of groups). [file 12917_2022_3532_MOESM3_ESM.docx]

**
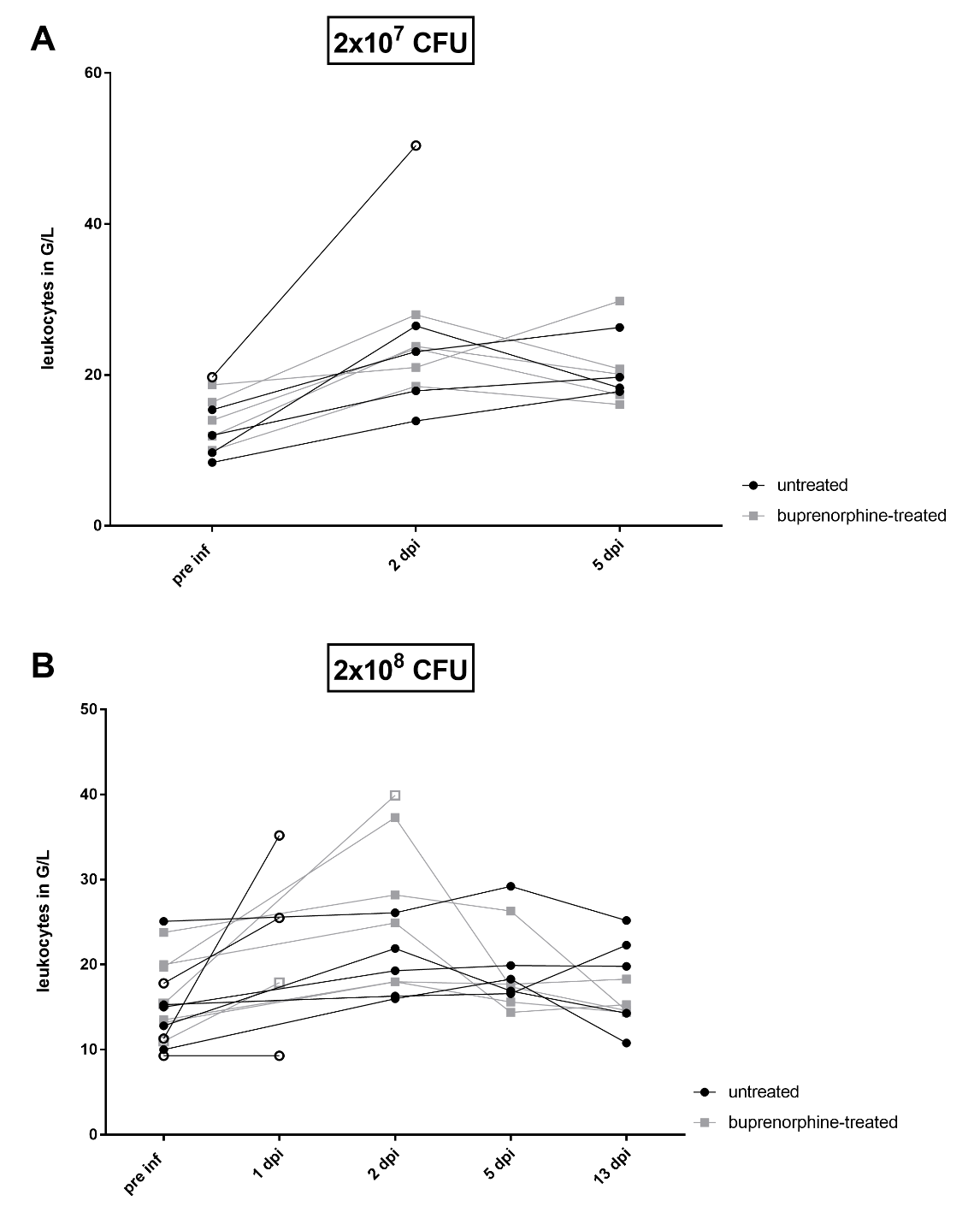
Additional File 3.** Blood leukocytes of piglets experimentally infected with *S. suis cps*3 strain 16667/3.

**Supplementary Fig. 1. Course of blood leukocytes in piglets w or w/o buprenorphine treatment after *S. suis cps*3 infection.** EDTA blood was taken before (pre inf) and after experimental infection with 2x10^7^ CFU (**A**) or 2x10^8^ CFU (**B**) of *S. suis* *cps*3 strain 16667/3. One group was treated i.m. with buprenorphine 0 to 5 days post infection (dpi). Unfilled symbols (**○** , **□**) indicate piglets prematurely euthanized due to reaching humane endpoints. Leukocytes increased after infection in all (**A**) and 14 of 15 (**B**) piglets, respectively, and no significant differences between buprenorphine-treated and untreated piglets were determined. Statistical analysis was conducted with the Mann-Whitney-*U*-test (comparison of groups).
