# Supplementary material for: A new S. suis serotype 3 infection model in pigs: lack of effect of buprenorphine treatment to reduce distress
Source: BMC Vet Res. 2022 Dec 12;18:435. doi: 10.1186/s12917-022-03532-w (PMC9743652; doi:10.1186/s12917-022-03532-w)
Supplement: Supplementary file 4 — Additional file 4: Supplementary Fig. 2. Survival of S. suis strains of the indicated cps in blood of BT and UT piglets (A and B) and survival of S. suis cps3 as well as specific serum IgM and IgG antibody levels in cps3 infected and non-infected piglets (C). [file 12917_2022_3532_MOESM4_ESM.docx]

**Additional File 4.** Bacterial survival of *S. suis* in porcine blood and serum antibody levels in *cps*3-infected and non-infected piglets.


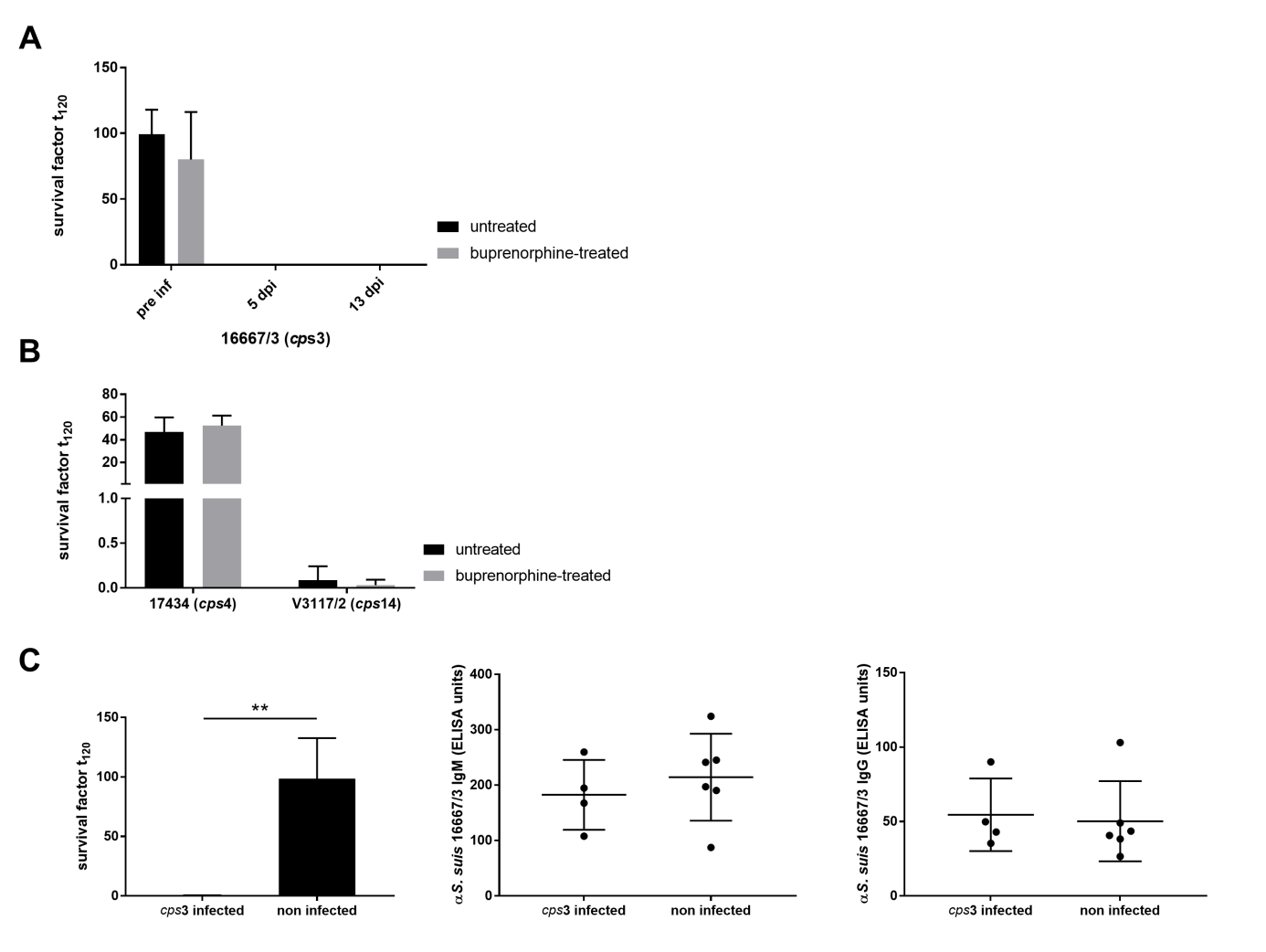


**Supplementary Fig. 2. Survival of *S. suis* strains of the indicated *cps* in blood of BT and UT piglets (A and B) and survival of *S. suis* *cps*3 as well as specific serum IgM and IgG antibody levels in *cps*3 infected and non-infected piglets (C).**  For collection of data shown in (A) and (B), blood was drawn pre infectionem (pre inf), 5 days post infection (dpi) and 13 dpi from piglets intravenously infected with 2x10^8^ CFU of *S. suis* *cps*3 strain 16667/3 (n = 5/ group). One group was treated i.m. with 0.05 mg/kg buprenorphine every 8 h 0 to 5 dpi as indicated. Survival of *S. suis cps*3 in porcine blood was determined at all mentioned time points (**A**), whereas survival of *S: suis* *cps*4 (17434) and *cps*14 (V3117/2) was only investigated 5 dpi (**B**). Strain 16667/3 (*cps*3) was efficiently killed in blood drawn 5 and 13 dpi (**A**). For data shown in (**C**), blood was collected 21 dpi from piglets infected with 2x10^7^ CFU of *cps*3 and non-infected littermates. Survival of *cps*3 and IgM as well as IgG serum antibodies binding to immobilized *cps*3 were determined (**C**). The survival factor represents the ratio of CFU at 120 min to CFU at time point zero. Survival factors above 1 indicate proliferation and below 1 killing of streptococci. Bars and error bars represent mean values and standard deviations, respectively. The Mann-Whitney-*U*-test was used for comparison of both groups. Significances are indicated (* *p* < 0.05, ** *p* < 0.01).
