# Supplementary material for: A new S. suis serotype 3 infection model in pigs: lack of effect of buprenorphine treatment to reduce distress
Source: BMC Vet Res. 2022 Dec 12;18:435. doi: 10.1186/s12917-022-03532-w (PMC9743652; doi:10.1186/s12917-022-03532-w)
Supplement: Supplementary file 5 — Additional file 5: Supplementary Fig. 3. Microbial adhesion of S. suis cps3, cps4 and cps14 to hydrocarbons. [file 12917_2022_3532_MOESM5_ESM.docx]

**Additional File 5.** Microbial adhesion of *S. suis cps*3, *cps*4 and *cps*14 to hydrocarbons (MATH).


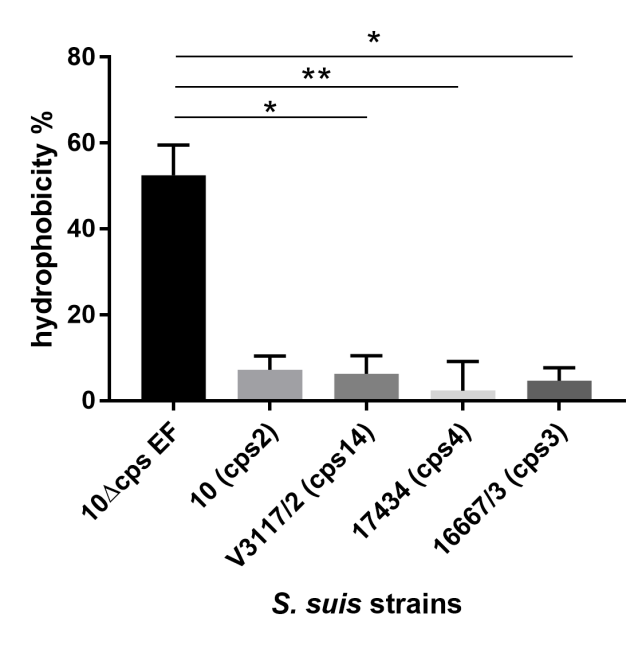


**Supplementary Fig. 3. Microbial adhesion of *S. suis* *cps*3, *cps*4 and *cps*14 to hydrocarbons.** Hydrophobicity of *S. suis* strains was determined after mixing of bacteria, resuspended in PBS, with hexadecane and incubation for 30min at room temperature [1]. Bars and error bars represent mean values and standard deviations, respectively. The One-Way ANOVA with a subsequent Dunn´s multiple comparisons test was used for comparison of strains. Significances are indicated (* *p* < 0.05, ** *p* < 0.01).

References

1. Öhlmann S, Krieger A-K, Gisch N, Meurer M, Buhr N de, Köckritz-Blickwede M von, et al. d-Alanylation of Lipoteichoic Acids in *Streptococcus suis* Reduces Association With Leukocytes in Porcine Blood. Front Microbiol. 2022;13:822369. doi:10.3389/fmicb.2022.822369.
