# Supplementary material for: A new S. suis serotype 3 infection model in pigs: lack of effect of buprenorphine treatment to reduce distress
Source: BMC Vet Res. 2022 Dec 12;18:435. doi: 10.1186/s12917-022-03532-w (PMC9743652; doi:10.1186/s12917-022-03532-w)
Supplement: Supplementary file 6 — Additional file 6: Supplementary Fig. 4. Serum cortisol in piglets infected with 2x10^7^ CFU S. suis cps3 w or w/o buprenorphine treatment. [file 12917_2022_3532_MOESM6_ESM.docx]

**
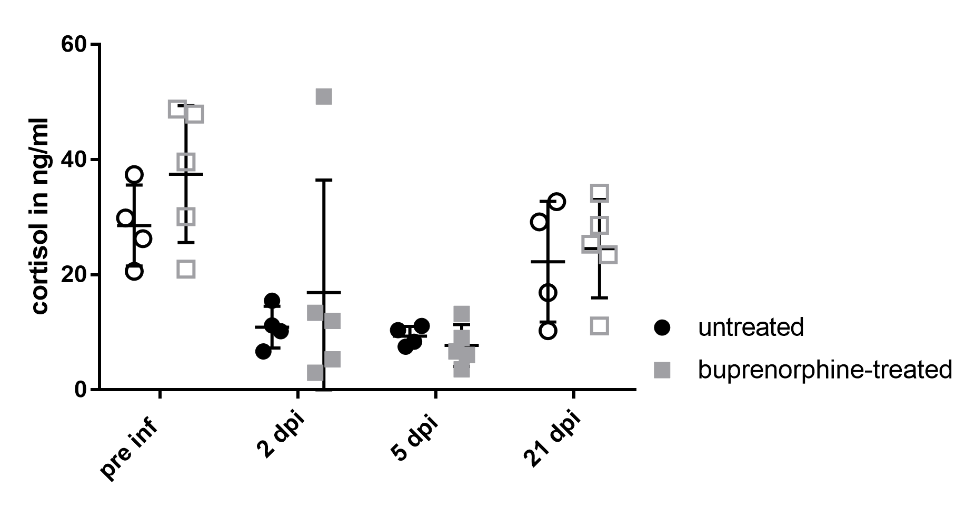
Additional File 6.** Serum cortisol levels of buprenorphine-treated and untreated piglets after experimental infection with 2x10^7^ CFU of *S. suis* strain 16667/3.

**Supplementary Fig. 4. Serum cortisol in piglets infected with 2x10^7^ CFU** ***S. suis* *cps*3 w or w/o buprenorphine treatment.** Blood of buprenorphine-treated (n = 5 ) (■) and untreated (n = 4) (●) piglets was taken before and at the indicated times after intravenous infection. As indicated, one group was treated i.m. with 0.05 mg/kg buprenorphine every 8 h 0 to 5 days post infection (dpi)**.** Unfilled symbols (**○** , **□**) represent serum cortisol levels determined in blood of anaesthetized piglets generally resulting in higher cortisol levels [1]. Prematurely euthanized piglets with clinical signs of severe disease were excluded. Statistical analysis was conducted with the Mann-Whitney-*U*-test (comparison of groups).

**References**

1. Daş G, Vernunft A, Görs S, Kanitz E, Weitzel JM, Brüssow K-P, Metges CC. Effects of general anesthesia with ketamine in combination with the neuroleptic sedatives xylazine or azaperone on plasma metabolites and hormones in pigs. Journal of animal science. 2016;94:3229–39. doi:10.2527/jas.2016-0365.
